# Liposomal-Naringenin Radiosensitizes Triple-Negative Breast Cancer MDA-MB-231 Cells *In Vitro*

**DOI:** 10.1049/2024/3786627

**Published:** 2024-06-28

**Authors:** Keenau Pearce, Samantha I. Cairncross, Mongi Benjeddou

**Affiliations:** Precision Medicine Unit Department of Biotechnology University of the Western Cape, Cape Town, South Africa

## Abstract

**Background:**

Naringenin has shown great promise in the realm of cancer therapeutics, demonstrating excellent cytotoxic action toward cancer cells and the enhanced effects of radiation therapy *in vitro*. However, the medicinal value of naringenin is severely limited clinically by poor bioavailability. Thus, multiple drug-delivery strategies for overcoming this limitation have been developed, of which liposomes are considered the most suitable due to their amphiphilic, modifiable, and biocompatible characteristics. In this study, we investigated the role of naringenin and liposomal-delivered naringenin as adjuncts to radiotherapy in the MDA-MB-231 triple-negative breast cancer cell line *in vitro*.

**Materials and Methods:**

Liposomal-naringenin was synthesized by thin-film hydration and extrusion and was characterized by spectrophotometry, dynamic light scattering, and zeta potential. The effects of free-from naringenin and liposomal-naringenin were evaluated toward MDA-MB-231 cell viability when combined with varying doses of radiation. Additionally, cell growth patterns, morphology, and colony formation were evaluated.

**Results:**

The analysis demonstrated IC_50_ values of 387.5 and 546.6 *µ*g/ml for naringenin and liposomal-naringenin, respectively. Naringenin and liposomal-naringenin significantly lowered cell viability, proliferation, and colony formation dose-dependently, as compared to radiation in isolation.

**Conclusion:**

The findings presented herein concur with previous accounts of the radiosensitizing potential of naringenin and further highlight the considerable biomedical application of liposomal-naringenin within the realm of radiotherapy.

## 1. Introduction

Breast cancer is one of the most frequently diagnosed malignant tumors affecting women on a global scale and is reported to be a significant contributor to cancer-related deaths. It typically arises due to changes in breast cells, particularly in those lining the ducts and the lobules, giving rise to ductal and lobular carcinoma, respectively [1, 2, 3]. Breast cancer may manifest in several patterns, including the triple-negative subtype, breast Paget disease, inflammatory breast cancer, non-Hodgkin lymphoma, and soft tissue carcinoma. While a direct cause for breast cancer is yet to be established, several risk factors have been identified and closely linked to its manifestation [2, 3]. These include age, sex, poor lifestyle choices, unfavorable dietary habits, and the resulting obesity, unfavorable environmental factors, social–psychological factors, and genetics [2, 3]. Particularly, recent reports have shown genetic mutation and family history to be involved in 5%–10% of breast cancer cases [3].

The global incidence and mortality of all cancers have seen rapid global growth, accounting for 19.3 million new cases and 10 million deaths in 2020 [1]. Of these, female breast cancer was the most frequently diagnosed and the 5th leading cause of cancer mortality worldwide, totaling 685,000 recorded deaths in 2020 [1]. In 2022, it was reported that female breast cancer accounted for 23.3 million new cases, comprising 11.6% of all cancer cases. It was further reported to be the 4th leading cause of cancer mortality worldwide, accounting for 6.9% of all cancer-related deaths [2, 4]. Moreover, female breast cancer was reported to account for approximately one in four cancer cases and one in six cancer deaths in women globally [2, 4]. The highest incidence rates were reportedly observed in France, Australia/New Zealand, Northern America, and Northern Europe [2, 4]. It is common for updated incidence and mortality data to trail behind the current year by up to 4 years due to the time needed for collection, analysis, and dissemination [3]. Considering the last available global cancer statistics were published in 2022, projections based on available data have been generated to gain insight into the current cancer burden [3]. For breast cancer in particular, projections for 2024 revealed a predicted 313,510 new cases diagnosed in women, along with 42,780 deaths in the United States alone [3]. Moreover, the triple-negative breast cancer subtype is estimated to comprise 20% of the global breast cancer-related cases [5, 6] and approximately 67% of all breast cancer mortalities [7]. Triple-negative breast cancer is a basal-like subtype characterized by the absence of progesterone and estrogen receptor expression, along with the absence of human epidermal growth factor 2 overexpression—owing to its aggressive, invasive, and recurrent nature, along with its resistance to treatment [7]. Radiotherapy is among the frequently utilized modalities for the treatment of multiple cancer types, including the lung, prostate, and breast [7, 8].

Radiotherapy exerts several effects on cancer cells, such as inducing DNA breaks, chromosomal rearrangement, generation of reactive oxygen species and free radical damage, and cytotoxicity [7]. However, there is inherent and acquired radioresistance in several cancers, such as triple-negative breast cancer [9], necessitating high radiation doses to achieve positive therapeutic outcomes. This ultimately increases the risk of damage to surrounding structures and, thus, the incurrence of unwanted side effects and decreased quality of life [10, 11]. In the case of breast cancer, reported side effects include dry, itchy, and discolored skin, fatigue, nerve damage, breast swelling and hardening, fatty tissue damage, fluid buildup, weakened bones of the sternum, and cardiovascular disease [11, 12]. Thus, the greatest limitation of radiotherapy, as a factor of dose, is the deleterious effects on otherwise healthy tissues. Therefore, overcoming the resistance is paramount to the mitigation of these unwanted side effects, while maintaining or indeed improving the therapeutic benefits of radiotherapy toward cancer [13, 14].

Naringenin is a flavonoid shown to exert a range of favorable biomedical effects, including cell cycle regulation, antioxidant activity, antiproliferation and carcinogenesis prevention, pro-apoptosis, and angio-inhibition [15, 16]. Moreover, naringenin was previously shown to significantly lower the resistance of A549 and NCI-H23 lung cancer cells and MCF-7 breast cancer cells toward the effects of radiation therapy *in vitro* [16, 17], demonstrating a potential application as a radiosensitizing agent—typically defined as compounds that, when combined with radiation, achieves greater tumor inactivation than otherwise would be expected [14]. However, the clinical usage of naringenin is severely limited due to poor water solubility, poor oral bioavailability and intestinal absorption, low stability, rapid metabolism, and ineffective transport across biological membranes, yielding low tumor site bioavailability [15, 18, 19, 20, 21]. While challenging, these limitations may be overcome through nanotechnology principles, such as liposomal delivery [22].

Liposomes, or lipid-based nanostructures, are the preferred nanocarriers for drug delivery systems owing to their amphiphilic nature, operable characteristics, and modifiable morphology, which includes particle size, encapsulation efficiency, zeta potential, and drug release profile [22, 23, 24, 25]. Their structure enables the entrapment of poorly soluble compounds, such as naringenin, inside its lipophilic bilayer, enhancing the stability, bioavailability, and drug targeting for improved therapeutic outcomes. With this considered, it stands to reason that liposomal delivery of naringenin may be a viable strategy for overcoming the aforementioned limitations while simultaneously maintaining or improving the anticancer and radiosensitizing effects of the flavonoid. Thus, this study aimed to investigate these parameters in the MDA-MB-231 triple-negative breast cancer cell line. The HaCaT human keratinocyte cell line was used as a representation of noncancerous tissue.

## 2. Materials and Methods

Unless otherwise noted, all chemicals and reagents were purchased from Sigma-Aldrich Chemical Company (St Louis, MO, USA) and Thermofisher Scientific (Waltham, MA, USA).

### 2.1. Preparation of Liposomal-Encapsulated Naringenin

Naringenin-encapsulated liposomes were prepared using the thin-film hydration method according to the previously described methodology with slight modifications [26]. In short, a 7 : 3 (w/w) of L-*α*-phosphatidylcholine and cholesterol was weighed out to a final weight of 25 mg, added into a round bottom flask, and dissolved with 15 ml methanol. NAR was subsequently added during the lipid phase at a concentration of 387.5 *µ*g/ml. The organic phase was evaporated under vacuum using a rotary evaporator at 45°C for 2-hr. The dried lipid film was hydrated with 5 ml distilled water and continuously swirled for ~30 min in a 40°C water bath. The solution was transferred into a clean 15 ml Greiner tube. The Avanti Polar Lipids, Inc. extruder was assembled, and the desired filter size (Whatman) was inserted and then placed on top of a heating block (Variomag Powertherm) heated to 50°C. The multilamellar liposome vesicles were extruded 15 times through each desired pore size polycarbonate filter ranging from 800 to 100 nm using Hamilton syringes. A total of 20 passages were performed for a single extrusion protocol. The resulting liposome vesicles encapsulating NAR (Lip-NAR) were stored at 4°C until further analysis. Liposomes were sterile-filtered using a 0.45 *μ*m syringe filter before experiments.

### 2.2. Characterization of Nanoparticles: Particle Size, Zeta Potential, and Encapsulation Efficiency

Encapsulation efficiency was determined by high-performance liquid chromatography coupled with mass spectrometry (HPLC–MS) according to the previously described methodology [26]. For size and charge analysis, the liposomal-encapsulated naringenin was suitably diluted with distilled water in a cuvette for analysis. The particle size and polydispersity index (PDI) were determined using dynamic light scattering via the ZetaSizer analyzer (Malvern). The solution was transferred from the cuvette to a folded capillary zeta cell, and the zeta potential of the liposomes was determined using the same instrument. The measured scattering intensities and zeta potential were analyzed using zeta-sizer software.

### 2.3. Cell Line and Culture

The MDA-MB-231 triple-negative breast cancer and the HaCaT human keratinocyte cell lines were acquired from the American Type Culture Collection (ATCC) and used for this study. These cells were cultivated at 37°C in 95% air and 5% CO_2_, following standard aseptic work procedures. MDA-MB-231 and HaCat cells were cultured in complete Dulbecco's Modified Eagle Medium (DMEM) supplemented with 10% fetal bovine serum and 1% penicillin (100 IU/ml) and streptomycin (100 *μ*g/m) in 25 cm^2^ culture flasks.

### 2.4. Irradiation of Cells

Cell cultures were irradiated with ^60^Co *γ*-rays using a Teletherapy unit (Theratron) at an approximate rate of 6 min and 20 s/1 Gy. In short, culture flasks were placed between a 5 cm thick Perspex plate to ensure dose buildup and a 5 cm backscatter plate with a dose rate of 0.1630 Gy/min for a 30 × 30 cm^2^ field size at 75 cm source to surface distance. The samples were exposed to radiation doses of 2, 4, and 8 Gy.

### 2.5. Cell Viability MTT Assay

The effect of naringenin and radiation toward MDA-MB-231 and HaCaT cell viability was determined using the 3- (4,5-dimethylthiazolyl-2)-2, 5-diphenyltetrazolium bromine (MTT) cell proliferation assay. Briefly, cells were seeded into 96-well polystyrene plates at 5 × 10^3^ cells/well in 100 *µ*l of complete growth medium and allowed to adhere. Next, cells were treated with varying concentrations (1,000, 500, 250, 125, 62.5, 31.25, and 15.125 *μ*g/ml) of naringenin solubilized in dimethylsulfoxide (DMSO), or liposomal-naringenin, respectively. DMSO did not exceed a final concentration of 0.5%. Each experiment included a vehicle control (VC). Based on the findings, a liposomal-naringenin concentration of 100 *μ*g/ml was selected for subsequent radiation studies, along with varying doses of radiation (2, 4, and 8 Gy).

Next, 10 *µ*l of MTT were added to each well, and plates were incubated at 37°C for an additional 4 hr. Growth medium and MTT were removed from each well, the remaining crystals were solubilized with 200 *µ*l of DMSO, and absorbance was measured at 570 nm using the SpectraMax M2 microplate reader (Molecular Devices, San Jose, USA). Results were expressed as percentage viability using the following formula: % viability = (*T*/*C*) × 100%, where *C* = absorbance of control and *T* = absorbance of treated cells. A total of six replicates were utilized for each treatment, and the data were expressed as an average of three independent experiments.

### 2.6. Morphological Analysis

To assess morphological changes in response to 2–8 Gy of radiation, the control and pretreated MDA-MB-231 and HaCaT cells were seeded in 96-well plates at a density of 5× 10^3^ cells/well in 100 *µ*l of complete DMEM and allowed to incubate for 24 hr. Cells were viewed using a Zeiss PrimoVert microscope, and subsequent images of the cells were captured using the ZEISS ZEN blue edition software (V 3.6).

### 2.7. Growth Curve Analysis

The proliferative properties of the MDA-MB-231 and HaCaT cell lines were examined by designing cell growth curves. The Trypan blue stain establishes cell viability based on the integrity of the cell membrane. The stain penetrates the dead cells and binds to the intracellular proteins to produce a distinctive blue color [27]. The untreated control and irradiated treated cells were seeded in 24-well plates at a density of 1 × 10^4^ cells/well and allowed to incubate for 24 and 48 hr. The cells were trypsinized following the incubation periods, respectively, and thoroughly resuspended. Next, trypan blue was added to the cell suspensions, and the cells were counted using a hemocytometer. Viable cell counts were determined as follows:

(1) $$\begin{array}{c} \text{Total viable cell count}\left(\text{cells/ml}\right)=\frac{\text{Total viable cell count}\times \text{Dilutionfactor}}{\text{The number of squares counted}}\times 10^{4}. \end{array}$$

 

### 2.8. Clonogenic Assay

The clonogenic assay is an *in vitro* cell survival assay formulated on the ability of a single cell to form a colony of at least 50 cells. This assay is performed to establish cell reproductive death following treatment with radiation, as well as the effectiveness of other cytotoxic agents [28]. Cells were seeded at densities of 200, 1,000, and 10,000 cells/well in 24-well plates for treatment with 0, 2–4, and 8 Gy, respectively. All subsequent findings were normalized. Following treatment and 10 days of incubation, media was removed, and cells were washed with phosphate-buffered saline. Next, colonies were fixed using an ice-cold 1 : 1 methanol: acetone solution for 15 min. Hereafter, the fixative was carefully removed, and colonies were stained using a 0.5% Crystal violet solution for 30 min. Following this, the crystal violet solution was removed, and each well was washed with distilled water. The plates were left to dry overnight before colonies were enumerated. Colonies greater than 50 cells were manually counted using a dissecting microscope with 10x magnification.

### 2.9. Statistical Analysis

Data were recorded and analyzed statistically using GraphPad Prism for Windows version 8.4.3 (GraphPad Software, San Diego, California, USA). Data were tested for normality and analyzed further using the independent *t*-test and one-way ANOVA. A *P*-value of less than 0.05 was considered significant.

## 3. Results

### 3.1. Liposome Characterization

Liposomes were characterized based on hydrodynamic size, PDI, and zeta potential, as summarized in Table 1.

Upon synthesis and extrusion, an average particle size of 98.52 nm with a PDI of 0.098 was measured for the empty liposomes. An average size of 95.39 nm was obtained for the liposomes loaded with naringenin (Lip-NAR), along with a PDI of 0.111. Following 6 months of storage at 4°C, an average size of 98.87 and 96.25 nm was obtained for the empty liposomes and liposomes loaded with naringenin, respectively. Average zeta potentials of −26.1 and −27.2 mV were measured for the unloaded and loaded liposomes, respectively. Following 6 months of storage at 4°C, average zeta-potentials of −25.9 and −26.8 mV were obtained for the empty liposomes and liposomes loaded with naringenin, respectively.

### 3.2. Cell Viability

#### 3.2.1. Naringenin and Liposomal-Naringenin

**Figure 1:**
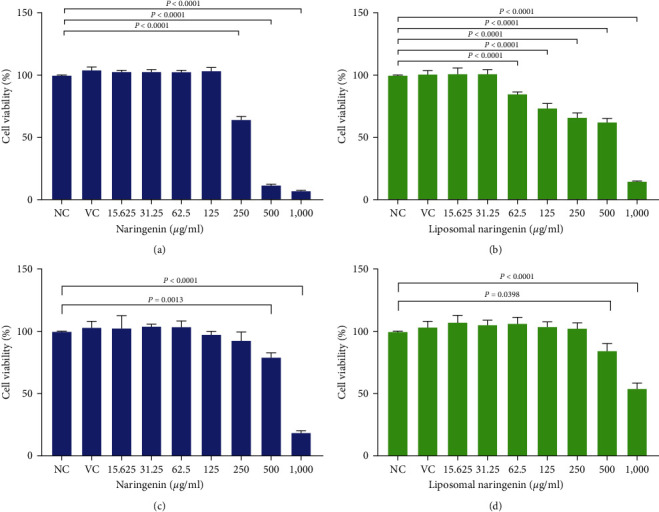
MDA-MB-231 (a and c) and HaCaT (b and d) cell viability as determined by the MTT assay over 24-hr. In MDA-MB-231 cells, as compared to the negative control (NC) naringenin (a) yielded significant (*P*  < 0.0001) decreases from 250 to 1,000 *µ*g/ml, respectively, and liposomal-naringenin (b) showed decreases in cell viability between control and 62.5–1,000 *µ*g/ml. One-way ANOVA revealed significant (*P*  < 0.0001) trends between the control and 1,000 *µ*g/ml for both naringenin and liposomal-naringenin, respectively. In HaCaT cells, naringenin (c) yielded significant (*P*=0.0013 and *P*  < 0.0001) decreases at 500 and 1,000 *µ*g/ml, respectively. Liposomal-naringenin (d) exhibited significant (*P*=0.0398 and *P*  < 0.0001) decreases at 500 and 1,000 *µ*g/ml, respectively. One-way ANOVA revealed significant (*P*  < 0.0001) trends between the control and 1,000 *µ*g/ml for both naringenin and liposomal-naringenin, respectively. VC demonstrated no toxicity in either cell line.

Following 24-hr exposure to naringenin (Figure 1(a)), significant (*P*  < 0.0001) increases in MDA-MB-231 cell viability were observed between 15.625 and 125 *µ*g/ml, followed by significant (*P*  < 0.0001) decreases from 250 to 1,000 *µ*g/ml, respectively. One-way ANOVA revealed significant (*P*  < 0.0001) trends between the control and 1,000 *µ*g/ml. Nonlinear regression analysis yielded a half-maximal inhibitory concentration (IC_50_) of 387.5 *µ*g/ml, with an *r*-square of 0.9367% and 95% CI of 217.7–748.9 *µ*g/ml (Table 2). Additionally, 0.5% DMSO used as the VC exhibited no toxicity. Subsequent 24-hr exposure of the MDA-MB-231 cells to liposomal-naringenin (Figure 1(b)) yielded significant (*P*  < 0.0001) decreases in cell viability between control and 62.5–1,000 *µ*g/ml. One-way ANOVA revealed significant (*P*  < 0.0001) trends between the control and 1,000 *µ*g/ml. Nonlinear regression analysis yielded an IC_50_ of 546.6 *µ*g/ml, with an *r*-square of 0.9024% and 95% CI of 459.6–646.9 *µ*g/ml. Moreover, the empty liposomes used as the VC yielded no deleterious effects on cell viability.

In HaCaT cells, 24-hr exposure to naringenin (Figure 1(c)), significant (*P*=0.0013) decreases in cell viability were observed at 500 *µ*g/ml, followed by a significant (*P*  < 0.0001) decreases at 1,000 *µ*g/ml, respectively. One-way ANOVA revealed significant (*P*  < 0.0001) trends between the control and 1,000 *µ*g/ml. Additionally, 0.5% DMSO used as the VC exhibited no toxicity. Subsequent 24-hr exposure of HaCaT cells to liposomal-naringenin (Figure 1(d)) demonstrated a significant (*P*=0.0398) decrease in cell viability at 500 *µ*g/ml, followed by a significant (*P*  < 0.0001) decrease at 1.000 *µ*g/ml. One-way ANOVA revealed significant (*P*  < 0.0001) trends between the control and 1,000 *µ*g/ml. No deleterious effects toward cell viability were yielded by the empty liposomes used as the VC.

#### 3.2.2. Radiation Combined with Naringenin and Liposomal Naringenin

**Figure 2:**
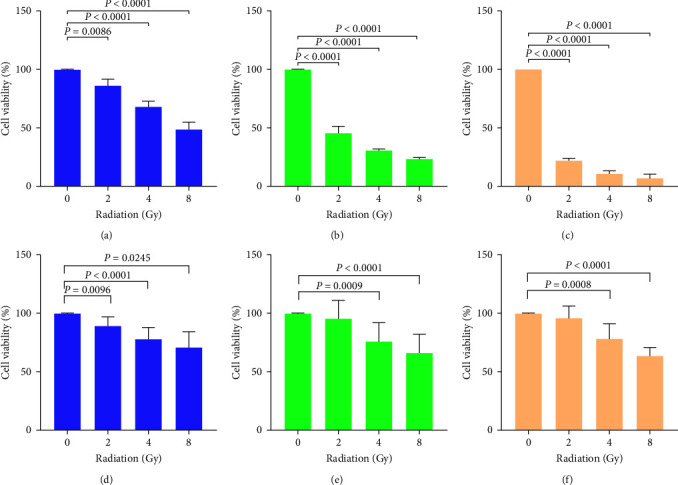
MDA-MB-231 (a–c) and HaCaT (d–f) cell viability, as determined by the MTT assay, in response to radiation in isolation, and radiation combined with 387.5 *µ*g/ml naringenin or 1,000 *µ*g/ml liposomal-naringenin, over 24-hr. In MDA-MB-231 cells, radiation in isolation (a) produced a significant (*P*=0.0086 and *P*  < 0.0001) decrease in cell viability, followed by significant (*P*  < 0.00001 and *P*  < 0.0001) decreases when combined with naringenin (b) and liposomal naringenin (c), respectively. One-way ANOVA exhibited a significant trend (*P*  < 0.0001) between the control and 8 Gy of radiation for each respective treatment group. In HaCaT cells radiation in isolation (d) produced significant (*P*=0.0009, *P*  < 0.0001, and *P*=0.0245) decreases in cell viability, along with significant (*P*=0.0009, *P*=0.0008, and *P*  < 0.0001) when combined with naringenin (e) and liposomal naringenin (f), respectively. One-way ANOVA exhibited a significant trend (*P*  < 0.0001) between the control and 8 Gy of radiation for each respective treatment group.

In MDA-MB-231 cells, the effects of radiation in isolation (Figure 2(a)), the combined effect of radiation and IC_50_ naringenin (Figure 2(b)), as well as radiation combined with IC_50_ Liposomal-naringenin (Figure 2(c)) toward cell viability was determined. Cells treated with radiation in isolation displayed significant decreases (*P*=0.0086 and *P*  < 0.0001) in cell viability at 2, 4, and 8 Gy, respectively. One-way ANOVA exhibited a significant trend (*P*  < 0.0001) between the control and 8 Gy of radiation. Naringenin combined with increasing doses of radiation decreased cell viability significantly (*P*  < 0.0001, *P*  < 0.0001, *P*  < 0.0001) at 2, 4, and 8 Gy, respectively. One-way ANOVA exhibited a significant trend (*P*  < 0.0001) between the control and 8 Gy of radiation. Similarly, the combined effects of liposomal-naringenin and increasing doses of radiation yielded significant (*P*  < 0.0001) reductions in cell viability between the control and 8 Gy of radiation. Additionally, one-way ANOVA demonstrated a significant trend (*P*  < 0.0001) between the control and 8 Gy.

In HaCaT cells, the effects of radiation in isolation (Figure 2(d)), the combined effect of radiation and IC_50_ naringenin (Figure 2(e)), as well as radiation combined with IC_50_ Liposomal-naringenin (Figure 2(f)) toward cell viability were determined. Cells treated with radiation in isolation displayed significant increases (*P*=0.0096, *P*  < 0.0001, and *P*=0.0245), in cell viability at 2, 4, and 8 Gy, respectively. One-way ANOVA exhibited a significant trend (*P*  < 0.0001) between the control and 8 Gy of radiation. Naringenin combined with increasing doses of radiation decreased cell viability significantly (*P*=0.0009 and *P*  < 0.0001) at 4 and 8 Gy, respectively. One-way ANOVA exhibited a significant trend (*P*  < 0.0001) between the control and 8 Gy of radiation. Similarly, the combined effects of liposomal-naringenin and increasing doses of radiation yielded significant (*P*=0.0008 and *P*  < 0.0001) reductions in cell viability at 4 and 8 Gy of radiation, respectively. Additionally, one-way ANOVA demonstrated a significant trend (*P*  < 0.0001) between the control and 8 Gy.

#### 3.2.3. Morphological Analysis

**Figure 3:**
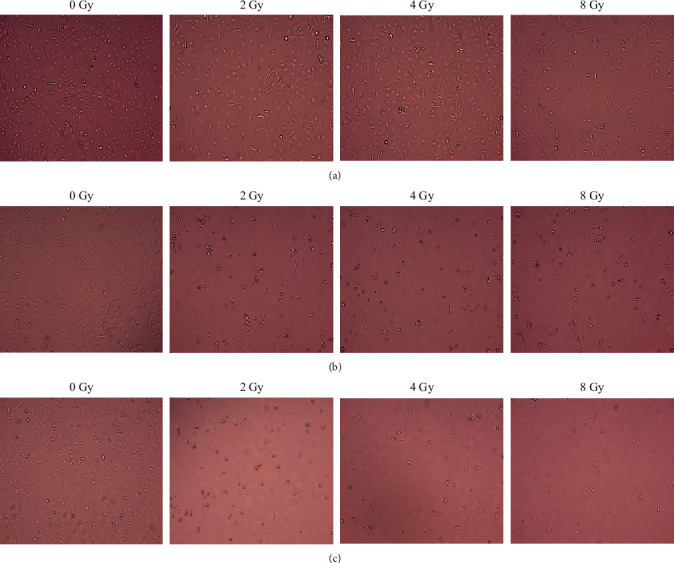
Morphological analysis of MDA-MB-231 cells following exposure to 2–8 Gy of radiation in isolation (a), and the combination of 2–8 Gy of radiation with 387.5 *μ*g/ml naringenin (b), or 2–8 Gy radiation with 546.6 *μ*g/ml liposomal-naringenin (c). As compared to radiation in isolation (a), significant alteration to expected epithelial-like cell morphology was observed when radiation was combined with naringenin (a) and liposomal-naringenin (b), yielding progressive cell rounding and signs indicative of cell death, respectively.

**Figure 4:**
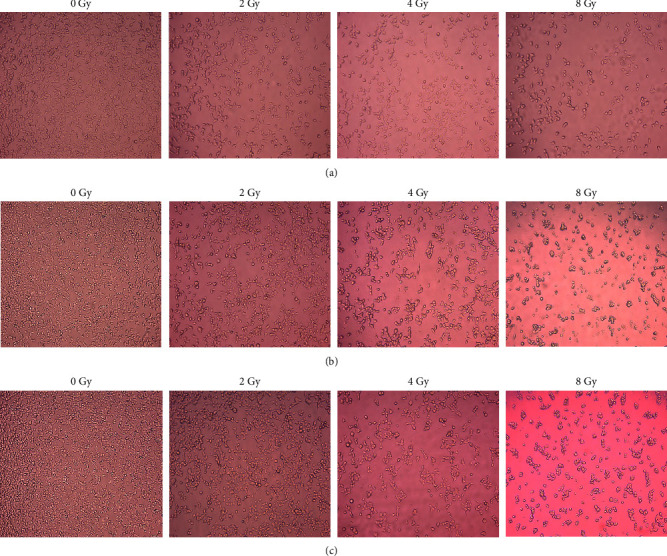
Morphological analysis of HaCaT cells following exposure to 2–8 Gy of radiation in isolation (a), and the combination of 2–8 Gy radiation with 387.5 *μ*g/ml naringenin (b), or 2–8 Gy radiation with 546.6 *μ*g/ml liposomal-naringenin (c). As compared to radiation in isolation (a), alterations to expected polygonal cell morphology, progressive cell rounding, and fewer observable cells were similarly observed when radiation was combined with naringenin (a) and liposomal-naringenin (b), respectively.

Following treatment of MDA-MB-231 cells (Figure 3) and HaCaT cells (Figure 4) with 2–8 Gy of radiation in isolation (Figures 3(a) and 4(a)), and the combined radiation with IC_50_ naringenin (Figures 3(b) and 4(b)) and IC_50_ liposomal-naringenin (Lip-NAR) (Figure 3(c) and 4(c)), cell morphology was examined and documented.

In MDA-MB-231 cells (Figure 3) treated with radiation in isolation (Figure 3(a)), no major deviations from the characteristic epithelial-like cell structure were observed following 2–8 Gy of radiation, with minor evidence of lowered cell number, cell swelling, and stress at 4–8 Gy. The combined effects of radiation with naringenin (Figure 3(b)) generated fewer observable cells at each radiation dose tested, with notably rounded cell morphology and cell scattering. Liposomal-naringenin (Figure 3(c)) yielded the lowest observable cell number, greatest alteration to cell morphology, and most prominent signs indicative of cell death.

In HaCaT cells (Figure 4) treated with radiation in isolation (Figure 4(a)), no major deviations from the characteristic polygonal cell structure were observed following 2–8 Gy of radiation, with an observably lowered cell number at 4–8 Gy. Signs of cell rounding and stress were observed at 8 Gy. The combined effects of radiation with naringenin (Figure 4(b)) generated fewer observable cells 4–8 Gy, with more rounded cell morphology and stress. Similarly, liposomal-naringenin (Figure 4(c)) yielded observably fewer cells at 4–8 Gy, along with rounded cell morphology at 8 Gy.

#### 3.2.4. Growth Curve Analysis

**Figure 5:**
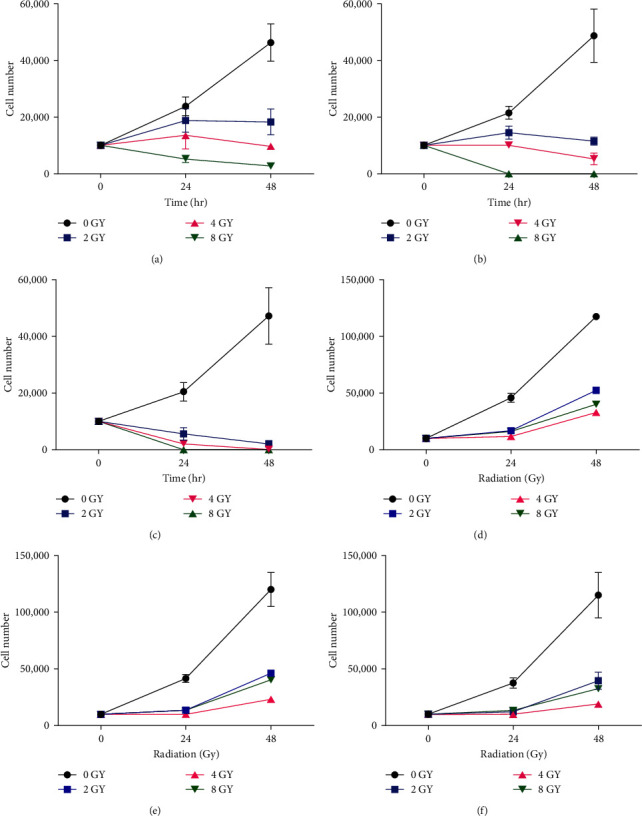
MDA-MB-231 (a–c) and HaCaT (d–f) cell growth in response to 2–8 Gy radiation in isolation (a and d) or combined with 387.5 *μ*g/ml naringenin (b and e) or 546.6 *μ*g/ml liposomal-naringenin (c and f) over 48 hr. As compared to control and radiation in isolation, the combined effects of radiation and naringenin (b) and liposomal-naringenin (c) notably suppressed MDA-MB-231 cell growth. In HaCaT cells, the combined effects of radiation and naringenin (c) and liposomal-naringenin (f) exhibited similar levels of cell growth inhibition.

Following treatment with radiation in isolation (Figure 5(a)) or combined with IC_50_ naringenin (Figure 5(b)) or IC_50_ liposomal-naringenin (Figure 5(c)), cell growth curves were determined over 48 hr in MDA-MB-231 (Figures 5(a), 5(b), and 5(c)) and HaCaT (Figures 5(d), 5(e), and 5(f)) cells. Average cell counts are reported.

Following treatment of MDA-MB-231 cells with radiation in isolation (Figure 5(a)), expected exponential increases in cell number were observed for control groups, accounting for 46,250 cells at 48 hr. Following treatment with 2 Gy of radiation, a slight increase in cell number was observed at 24 hr, totaling 18,750 cells, followed by a slight decline at 48 hr, totaling 18,312 cells. Similarly, treatment with 4 and 8 Gy of radiation yielded notably low cell numbers of 13,250 and 5,250 cells at 24 hr, respectively, followed by slight declines at 48 hr, totaling 9,687 and 2,750 respective cells. Cells treated with 4 and 8 Gy of radiation exhibited the lowest observable cell numbers. In HaCaT cells (Figure 5(d)), an exponential growth pattern was observed in controls, accounting for 117,500 cells at 48 hr. Following treatment with 2–8 Gy of radiation, 16,250, 14,250, and 12,150 respective cells were counted at 24 hr. At 48 hr, cell counts increased to 52,500 and 40,206 respective cells at 2 and 4 Gy. A notably smaller increase was observed in cells treated with 8 Gy, totaling 33,125 cells.

Hereafter, the effect of radiation combined with naringenin on the growth curve was determined over 48 hr (Figure 5(b)). Exponential cell growth was observed for control groups as expected, accounting for 48,750 cells at 48 hr. Following treatment with 2 and 4 Gy of radiation, a slight increase in cell number was observed at 24 hr, totaling 14,562 and 11,000 respective cells, followed by sharp declines at 48 hr, accounting for 11,625 and 5,200 respective cells. At 8 Gy of radiation, cells were no longer detectable at 24 and 48 hr, respectively. In HaCaT cells (Figure 5(e)), the expected exponential growth pattern was observed for the control, accounting for 122,055 cells at 48 hr. Following treatment with 2–8 Gy of radiation, 13,250, 12,118, and 10,050 respective cells were counted at 24 hr. At 48 hr, cell counts increased to 46,201 and 41,271 respective cells at 2 and 4 Gy. A notably smaller increase was observed in cells treated with 8 Gy, totaling 23,052 cells.

Next, the combined effects of radiation and liposomal-naringenin on the growth curve were determined over 24 and 48 hr (Figure 4(c)). Exponential cell growth was observed for control groups as expected, accounting for 47,255 cells at 48 hr. Following treatment with 2 Gy of radiation, a sharp decrease in cell number was observed at 24 hr, totaling 5,625 cells, followed by a further decline at 48 hr averaging 5,625 cells. Similarly, treatment with 4 Gy of radiation yielded markedly lower cell numbers at 24 hr, totaling 2,125 cells, followed by the total absence of observable cells at 48 hr. A similar absence of observable cells was observed at 8 Gy of radiation over 24 and 48 hr, respectively. In HaCaT cells (Figure 5(f)), the expected exponential growth pattern was observed for the control, accounting for 115,055 cells at 48 hr. Following treatment with 2–8 Gy of radiation, 14,055, 11,265, and 10,550 respective cells were counted at 24 hr. At 48 hr, cell counts increased to 39,501 and 32,551 respective cells at 2 and 4 Gy. A notably smaller increase was observed in cells treated with 8 Gy, totaling 19,102 cells.

#### 3.2.5. Colony Formation and Counts

**Figure 6:**
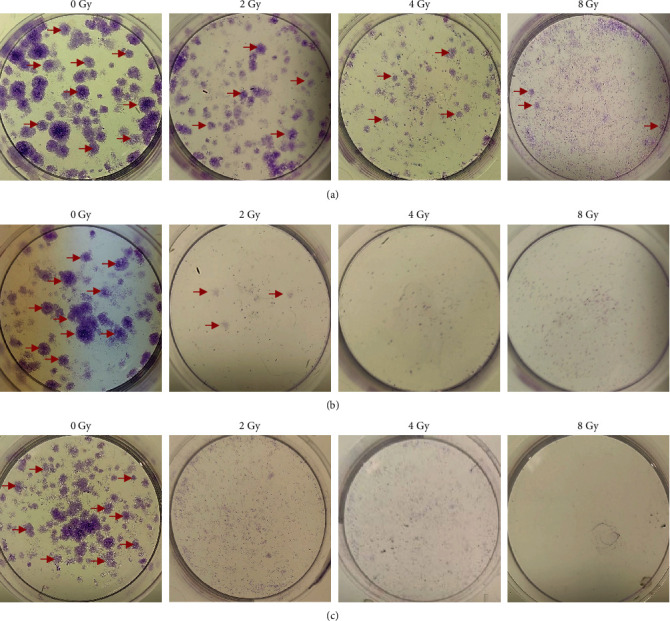
MDA-MB-231 colony formation over 10 days following exposure to 2–8 Gy radiation in isolation (a), 2–8 Gy radiation combined with 387.5 *μ*g/ml naringenin (b), and 2–8 Gy radiation combined with 546.6 *μ*g/ml liposomal-naringenin (Lip-NAR) (c). The progression of colony number is illustrated by the red arrows. As compared to the control (0 Gy), an expected stepwise reduction in formed colonies was observed in cells treated with radiation in isolation (2–8 Gy), followed by a significant reduction in observable colonies when combined with naringenin (2–8 Gy), and no colonies when combined with liposomal naringenin (2–8 Gy).

**Figure 7:**
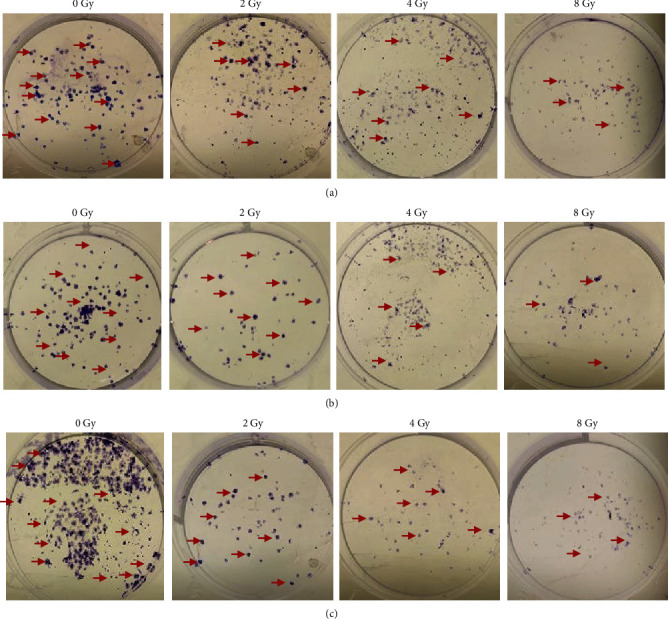
HaCaT colony formation over 10 days following exposure to 2–8 Gy of radiation in isolation (a), 2–8 Gy of radiation combined with 387.5 *μ*g/ml naringenin (b), and 2–8 Gy radiation combined with 546.6 *μ*g/ml liposomal-naringenin (Lip-NAR) (c). The progression of colony number is illustrated by the red arrows. As compared to control (0 Gy), an expected stepwise reduction in formed colonies was observed in cells treated with radiation in isolation (2–8 Gy), followed by a similar overall reduction in observable colonies when combined with naringenin (2–8 Gy), and liposomal naringenin (2–8 Gy).

Following treatment with 2–8 Gy of radiation, colony formation, and colony number were determined over 10 days in MDA-MB-231 (Figure 6) and HaCaT cells (Figure 7).

**Figure 8:**
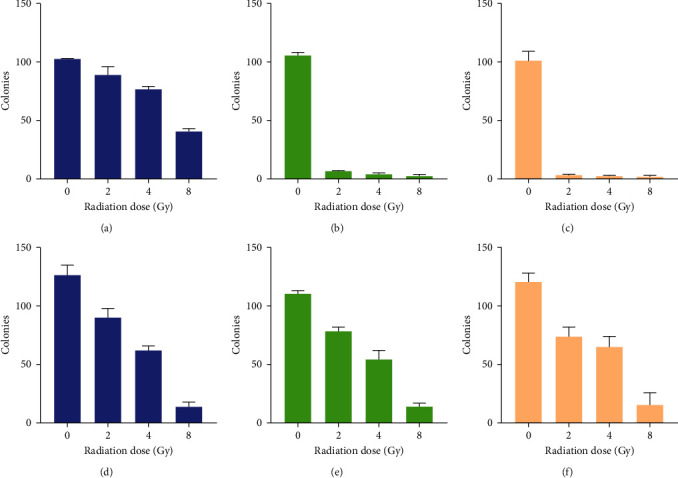
Colony counts in MDA-MB-231 (a–c) and HaCaT (d–f) cells 10-days posttreatment with 2–8 Gy of radiation in isolation and 2–8 Gy of radiation combined with 387.5 *μ*g/ml naringenin and 546.6 *μ*g/ml liposomal-naringenin. In MDA-MB-231 cells, an expected dose-dependent reduction in colony counts was observed in cells treated with 2–8 Gy radiation only (a), followed by significantly fewer observable colonies when combined with naringenin (b), and the complete absence of colonies when combined with liposomal-naringenin (c). In HaCaT cells, a similar stepwise reduction in observable colonies was observed for 2–8 Gy of radiation only (d) and 2–8 Gy of radiation combined with naringenin (e) and liposomal-naringenin (f).

In MDA-MB-231 cells (Figure 6), the clonogenic assay revealed a stepwise reduction in observable colonies between the untreated control groups, 0, and 8 Gy of radiation (Figure 6(a)). Colony counts (Figure 8) revealed an average of 102 colonies for the untreated controls, followed by 89 colonies at 2 Gy, 76 colonies at 4 Gy, and 40 colonies at 8 Gy (Figure 8(a)). In HaCaT cells (Figure 8), the clonogenic assay similarly revealed a stepwise reduction in observable colonies between 0 and 8 Gy of radiation (Figure 8(a)), with average colony counts of 126 for 0 Gy, 90 colonies at 2 Gy, 62 colonies at 4 Gy, and 14 colonies at 8 Gy (Figure 8(d)).

Next, the combined effect of IC_50_ naringenin and 2−8 Gy of radiation toward colony formation (Figure 6(b)) and number (Figure 6(b)) were determined. In MDA-MB−231 cells, the clonogenic assay revealed few observable colonies at each dose of radiation tested (Figure 6(b)). Colony counts revealed an average of 105 colonies for the untreated controls, followed by 7 colonies at 2 Gy, 4 colonies at 4 Gy, and 2 colonies at 8 Gy (Figure 8(b)). In HaCaT cells (Figure 7), the clonogenic assay similarly revealed a stepwise reduction in observable colonies between 0 and 8 Gy of radiation (Figure 7(a)), with average colony counts of 110 for 0 Gy, 78 colonies at 2 Gy, 54 colonies at 4 Gy, and 12 colonies at 8 Gy (Figure 8(e)).

Hereafter, the combined effect of IC_50_ liposomal-naringenin and 2–8 Gy of radiation towards colony formation (Figure 6(c)) and colony number (Figure 6(c)) were determined. In MDA-MB-231 cells, the clonogenic assay revealed no observable colonies at each dose of radiation tested (Figure 6(c)). Colony counts revealed an average of 101 colonies were obtained for the untreated controls (Figure 8(c)). In HaCaT cells (Figure 7), the clonogenic assay similarly revealed a stepwise reduction in observable colonies between 0 and 8 Gy of radiation (Figure 7(a)), with average colony counts of 120 for 0 Gy, 74 colonies at 2 Gy, 65 colonies at 4 Gy, and 16 colonies at 8 Gy (Figure 8(f)).

## 4. Discussion

Naringenin possesses enormous medicinal potential, ranging from being anticarcinogenic and anti-inflammatory to being radiosensitizing [15, 16]. However, several factors limit its clinical usage, such as poor water solubility, poor oral bioavailability, and low tumor site bioavailability [15, 18, 19, 20, 29]. These limitations must be overcome to fully realize the vast biomedical potential of the flavonoid—a challenge feasibly overcome through nanocarrier systems. Nanocarrier systems are confirmed to greatly enhance the pharmacological potential of poorly bioavailable flavonoids, such as naringenin, and thus, they are frequently explored in the effort to mitigate their biomedical limitations. To this end, liposomal delivery of naringenin has become a particularly favorable approach due to its unique and modifiable characteristics [22, 23, 24, 25, 30, 31]. Considering the radiosensitizing potential of naringenin and the implications of nanocarriers, this study aimed to investigate liposome-delivered naringenin, in contrast to the free-form flavonoid, as a potential adjunct to radiotherapy. These effects were assessed by cell morphology, viability and growth curve, and colony formation in MDA-MB-231 and HaCaT cells.

As compared to MDA-MB-231 cells treated with radiation in isolation, naringenin, and liposomal-naringenin resulted in a higher degree of morphological changes consistent with cell death, such as rounded and floating cells, at each dose of radiation tested, with the liposomal-naringenin resulting in the greatest observable cell death. These observations indicate that naringenin enhanced the effects of radiation on these cells, adding validity to previous accounts of the radiosensitizing activity of the flavonoid [16, 32]. Importantly, liposomal-naringenin notably exceeded the capabilities of the free-form flavonoid, adding validity to the use of nanocarriers as a viable strategy to overcome the inherent biomedical limitations. Furthermore, the findings presented here are consistent with previous accounts demonstrating more favorable cell death when radiation was combined with nanodelivered naringenin in particular [16, 32]. It is important to note that the combination of radiation with naringenin and liposomal-naringenin had no greater effect on HaCaT cells, as compared to the effects of radiation in isolation, suggesting a degree of specificity toward MDA-MB-231 cells. Nevertheless, the present effects on cell growth, viability, and colony formation may further support the compound's antiproliferative and evident radiosensitizing potential.

Growth patterns of MDA-MB-231 cells were notably altered in a dose and time-dependent manner in groups treated with radiation in isolation, whereby 4 and 8 Gy displayed the greatest reduction in cell number following 48 hr of growth—an occurrence accompanied by a significant dose-related decrease in cell viability. When radiation was combined with naringenin, however, further reductions in cell number were observed at 2 and 4 Gy, followed by a complete absence of detectable cells at 8 Gy. In addition, significant dose-dependent reductions in cell viability were also observed at each radiation dose tested. Furthermore, these effects toward cell growth and viability were strengthened through the combined effects of radiation and liposomal-naringenin. Similarly, the adjunct use of naringenin and liposomal-naringenin exceeded the capabilities of radiation in isolation toward colony formation. Interestingly, these enhanced effects were not observed in HaCaT cells.

These findings are consistent with previous studies demonstrating the ability of naringenin to inhibit colony formation in multiple cell lines, including the MCF-7 and MDA-MB-231 breast cancer cells [33, 34, 35, 36]. Based on the present findings, it is clear that naringenin improved the destructive effects of radiation toward the MDA-MB-231 cell growth, viability, and colony formation, thus supporting previous accounts of the flavonoid's antiproliferative and radiosensitizing potential [16, 32]. Moreover, liposomal delivery of naringenin considerably enhanced the deleterious effects of radiation on cell growth, viability, and colony formation, as compared to the free-form flavonoid. These findings may further support liposomal delivery of naringenin as a simple strategy to simultaneously overcome the aforementioned limitations surrounding the flavonoid, while also creating new therapeutic avenues within the realm of radiotherapy [15, 18, 19, 20].

It must be mentioned, however, that while this study provides valuable insight into the potential application of liposomal-naringenin, it is subject to several limitations. These include the limited scope of assays that were performed, ultimately hindering the elucidation of the precise mechanistic action of these nanoparticles. Moreover, the cellular incorporation rate of these nanoparticles was not determined and investigating this aspect would add valuable insight to future applications. Finally, it was beyond the scope of this study to determine the effect of these nanoparticles on noncancerous breast cells. Thus, further investigation addressing these limitations is essential.

## 5. Conclusions

It may be concluded that naringenin indeed possesses substantial application within the realm of radiotherapy owing to its sensitizing properties toward cancer cells. Moreover, the findings presented here may support the use of nanocarried naringenin to overcome the clinical limitation of the flavonoid. Considering the medical value of the findings presented herein, liposomal naringenin is likely to have considerable biomedical applications as a management strategy for breast cancer and cancer overall.

## Figures and Tables

**Table 1:** Average size, PDI, and zeta potential of synthesized empty and loaded liposomes.

Measurement period	Sample	Size (nm)	PDI	Zeta potential (mV)	Encapsulation efficiency (%)
At synthesis	Empty liposomes	98.52	0.098	−26.1	—
	Lip-NAR	95.39	0.111	−27.9	96

6 months after synthesis	Empty liposomes	98.87	0.108	−25.9	—
	Lip-NAR	96.25	0.132	−26.8	—

At synthesis, empty liposomes had an average size of 98.52 nm, PDI of 0.098, and zeta potential of −26.1 mV. Loaded liposomes (Lip-NAR) had an average size of 95.39 nm, PDI of 0.111, and zeta potential of −27.9 mV. At 6 months after synthesis, empty liposomes had an average size of 98.87 nm, PDI of 0.108, and zeta potential of −25.9 mV. Loaded liposomes (Lip-NAR) had an average size of 96.25 nm, PDI of 0.132, and zeta potential of −26.8 mV. PDI, polydispersity index; mV, millivolts, nm: nanometers.

**Table 2:** Half maximal inhibitory concentration (IC_50_) analysis for naringenin and liposomal-naringenin (Lip-NAR) in MDA-MB-231 cells over 24 hr of exposure.

Sample	NAR_IC50_ (*µ*g/ml)	95% CI (*µ*g/ml)	*r*-Squared
Naringenin	387.5	217.7–748.9	0.9367
Lip-NAR	546.6	459.6–646.9	0.9024

Naringenin demonstrated an IC_50_ of 387.5 *µ*g/ml (95% CI: 217.7 −748.9, *r*^2^ = 0.9367). Lip-NAR exhibited an IC_50_ of 549.6 *µ*g/ml (95% CI: 459.6–646.9, *r*^2^ = 0.9024). CI: 95% confidence interval.

## Data Availability

Data will be made available upon request from Keenau Pearce: kpearce@uwc.ac.za.
